# Medical Students’ Perceptions at Dow University of Health Sciences using Dundee Ready Education Environment Measure Inventory

**DOI:** 10.12669/pjms.40.4.7712

**Published:** 2024

**Authors:** Bader Faiyaz Zuberi, Majid Ahmed Shaikh, Faisal Faiyaz Zuberi, Tazeen Rasheed, Faiza Sadaqat Ali, Zunaira Nawaz

**Affiliations:** 1 Prof. Bader Faiyaz Zuberi, FCPS, Meritorious Professor (Rtd) Gastroenterologist OMI Hospital, Karachi, Pakistan; 2 Dr. Majid Ahmed Shaikh, FCPS, Assistant Professor, Department of Medicine, Dow Medical College, Karachi, Pakistan; 3 Prof. Faisal Faiyaz Zuberi, FCPS, Ojha Institute of Chest Diseases, DUHS, Karachi, Pakistan; 4 Dr. Tazeen Rasheed, FCPS,Associate Professor, Department of Medicine, Dow Medical College, Karachi, Pakistan; 5 Dr. Faiza Sadaqat Ali, FCPS,Senior Registrar, Department of Medicine, Dow Medical College, Karachi, Pakistan; 6 Dr. Zunaira Nawaz, FCPS,Assistant Professor, Department of Medicine, Dow Medical College, Karachi, Pakistan

**Keywords:** DREEM Inventory, Curriculum, Educational Environment

## Abstract

**Objectives:**

To assess medical students’ perceptions regarding learning, teachers, academics, atmosphere at campus and social self-perceptions at Dow University of Health Sciences using Dundee Ready Education Environment Measure (DREEM) Inventory.

**Method:**

This cross-sectional observational study was conducted from March 1^st^, 2022 to September 30^th^, 2022. All medical students at Dow University of Health Sciences were offered to participate. All students were given the choice to respond to DREEM questionnaire via online Form or printed copy anonymously. The DREEM Inventory measures five domains of students’ perceptions of a given institution. Comparison of responses between different years and institution was carried out by χ^2^ test. Means scores were compared by Student’s t-test and ANOVA, p-value of ≤.05 was taken as significant.

**Results:**

Total of 1054 out of 1750 (60.23%) students submitted fully completed forms and were included, out of these 632 (60.0%) belonged to Dow Medical College & 422 (40.0%) belonged to Dow International Medical College. The mean ±SD of total score of DREEM by DMC students was 100.07 ±31.46 and that of DIMC was 100.52 ±32.73. According to DREEM Global scoring both colleges scores fell into category of “many problems”. Analysis according to domains showed that maximum score was given to 2^nd^ domain of their “perception regarding teachers” and minimum score was allocated to 5^th^ domain regarding “social self-perceptions”.

**Conclusion:**

Overall Students perceived environment at DUHS as “many problems” category. This needs to be investigated for betterment of Educational Environment (EE) at campus.

## INTRODUCTION

Educational Environment (EE) at campus is an important factor in students’ learning and development. These include many factors, e.g., curriculum, teachers’ attitudes, self-perception, campus environment and social perceptions etc. Curriculum is defined as the process of learning during the period of student educational process and the document is planned according to the goals of institution.^1^ This process includes not only the syllabus, but the overall factors mentioned above. Assessment of the needs of targeted learners is one of the primary objectives of curriculum development. Curriculum should meet the needs of its learners; it is either inefficient or ineffective if it fails to meet this goal. The feedback refers to the information described by the students for the performance in each activity that is intended to guide their future performance in the course they taught.^2^,^3^. Medical programs offer a systems-based curriculum in which learning is integrated and there is a system to assess the strength and weakness of the curricula by a mechanism called Dundee Ready Education Environment Measure (DREEM) inventory. The DREEM Inventory has been validated with high reliability in many countries to assess the educational environment of health professional/medical schools and has been translated to Spanish too.^4^

Dow University started developing the integrated curricula in 2009 and implemented it in 2011. Up to now six batches have passed out with this curriculum.^5^ The new curricula is designed to have both horizontal and vertical integration in all subjects taught during the course of MBBS. Assessment of education environment by an inventory is a healthy tool to determine the effectiveness of curriculum. One study carried out in 2013 in DUHS for assessment of educational environment showed total mean score of 57.2%, at that time Sind Medical College was also affiliated with DUHS.^6^ Much time has passed and there is need to reassess the students’ perception of educational environment again. It is therefore important to identify the elements operating in institution and students’ perception about them to form foundations for modifications that would augment students’ understandings in relation to curriculum teaching goals.

This exercise will help to identify Medical Students’ Perceptions at Dow University of Health Sciences (DUHS) using Dundee Ready Education Environment Measure (DREEM) Inventory and will thus help to improve it for better teaching and learning.

## METHODS

This cross-sectional Observational study was conducted between March 1^st^, 2022 to September 30^th^, 2022. Study was conducted simultaneously in two medical colleges, Dow Medical College (DMC) & Dow International Medical College (DIMC) that are affiliated with Dow University of Health Sciences (DUHS). All students at both colleges were offered to participate.

### Study Instrument

DREEM questionnaire is a 50-items questionnaire where each item is scored using a 5-point Likert scale between 0 to 4 (4 = very much agree; 3 = agree; 2 = not sure; 1 = disagree; 0 = strongly disagree). Reverse coding is required for 9 items (4, 8, 9, 17, 25, 35, 39, 48, and 50 [meaning that 4 = strongly disagree; 3 = disagree; 2 = not sure; 1 = agree; 0 = very much agree]).^7^

The total scale consists of 5 domains:

### Ethical approval

It was taken from IRB of DUHS vide letter number IRB-2363/DUHS/Approval/2022/714 dated 2-2-2022.

1. Students’ Perceptions of Learning: 12 items (maximum score, 48)
2. Students’ Perceptions of Teachers: 11 items (maximum score, 44)
3. Students’ Academic Self-Perceptions: 8 items (maximum score, 32)
4. Students’ Perceptions of Atmosphere: 12 items (maximum score, 48)
5. Students’ Social Self-Perceptions: 7 items (maximum score, 28)

Items with mean scores of ≥3.5 are considered true positive points, while those ≤2.0 are considered negative or problem areas. The rest are considered as areas where improvements can be achieved by addressing the situation. The maximum global score of DREEM is 200 and is interpreted as under:^8^

Very Poor <50

Many Problems 50-100

More Positive than Negative 101-150

Excellent. 151-200

### Inclusion criteria:

- Students of DUHS enrolled in MBBS program.

### Exclusion criteria:

- None.

### Data Collection

All students of DMC & DIMC from all academic years were offered the chance to fill in proforma anonymously. Both online Google forms and their printed hard copies were created and were offered to students for participation by any method they choose. Online forms were forwarded by WhatsApp/Email using the college student database of mass email server after permission form relevant principals. Information regarding URL of online form was also displayed after lectures in classes and questionnaire along with a respondent information sheet, was handed to all students present in the class (each semester separately) after routine lectures. The information sheet included a short introduction to the objectives of the study and of DREEM. Since it was anonymous, a separate consent Form was not collected. If questionnaires were returned filled, consent was implicit; if questionnaires were returned blank, non-consent was considered. The data was handled and stored in accordance with the tenets of the Declaration of Helsinki (1964, seventh revision in 2013).^9^ The submitted forms were then entered/imported in SPSS for analysis.

### Data Analysis

The DREEM Inventory measures five domains or subscales of students’ perceptions of a given institution. These include students’ perceptions regarding learning, teachers, academic self-perception, atmosphere at campus and social self-perceptions. Total environment score and scores for the five domains or subscales were calculated. Comparison of responses between different years and institution was carried out by χ^2^ test. Means scores were compared by Students’ t-test and ANOVA, *p*-value of ≤.05 was considered significant.

## RESULTS

A total of 1054 students returned/submitted fully completed forms and were included in the study, out of these 632 (60.0%) belonged to DMC & 422 (40.0%) belonged to DIMC. In DMC 459 (72.6%) were females & 173 (27.4%) were males, while in DIMC 256 (60.7%) were females & 166 (39.3%) were males. The year-wise distribution of students who responded is detailed in Table-I.

**Table-I:** Year wise distribution of students who responded as per their college affiliation.

	College		Total

	DMC n (%)	DIMC n (%)	
1st Year	62 (9.8)	91 (21.6)	153 (14.5)
2nd Year	180 (28.5)	95 (22.5)	275 (26.1)
3rd Year	159 (25.2)	88 (20.9)	247 (23.4)
4th Year	87 (13.8)	85 (20.1)	172 (16.3)
5th Year	144 (22.8)	63 (14.9)	207 (19.6)

Total	632	422	1054

Kindly write n (%) in one row instead of two.

The mean ±SD of total score of DREEM by DMC students was 100.07 ±31.46 and that of DIMC was 100.52 ±32.73, there was no significant difference in scores given by students of two colleges regarding curriculum [t (1052) = -.222; *p* = .824]. According to DREEM Global scoring both colleges scores fell into category of “many problems”. Year wise distribution of means of DREEM Global scores showed that in both colleges’ year one, two and three responses were in category “more positive than negative” but year four & five responses were in category “many problems”. Year wise mean DREEM Global scores are given in Table-I. Students DREEM scores according to gender showed that males gave higher scores as compared to females (101.72 ±32.2 vs 99.56 ±31.8), but the difference was not statistically significant [t (1052) = 1.03; *p* =.306]. Table-II

**Table-II:** Year wise DREEM Scores of DMC & DIMC Students.

	DMC	DIMC	Total
	Mean ±SD	Mean ±SD	Mean ±SD
1st Year	103.61 ±26.63	113.02 ±34.81	109.21 ±32.00
2nd Year	109.55 ±33.93	89.39 ±30.36	102.59 ±34.07
3rd Year	101.17 ±28.81	107.23 ±32.31	103.33 ±30.18
4th Year	89.34 ±26.55	93.09 ±28.40	91.20 ±27.47
5th Year	91.98 ±32.04	99.90 ±31.65	94.39 ±32.05

Total	100.07 ±31.46	100.52 ±32.73	100.25 ±31.96

**Fig.1:**
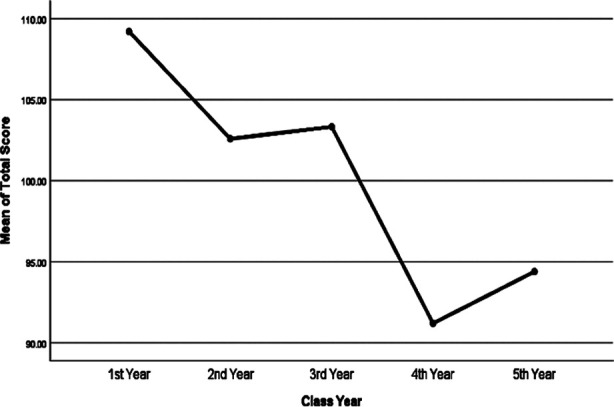
Means plot of inter-class difference in DREEM scores.

Minimum scores were given by 4^th^ year students, while maximum scores were given by 1^st^ year students. Inter-class difference of scores was assessed by one-way ANOVA test which showed significant differences in scores between classes except that between 2^nd^ and 3^rd^ years, details are given in Table-III and Fig.1.

**Table-III:** Comparison of inter-class difference in DREEM scores by one-way ANOVA.

(I) Class Year	(J) Class Year	Mean Diff. (I-J)	SE	Sig.	95% CI	

					Lower Bound	Upper Bound
1^st^ Year	2^nd^ Year	6.62*	3.17	.037	0.40	12.85
	3^rd^ Year	5.88	3.24	.070	-0.47	12.23
	4^th^ Year	18.01**	3.50	<.001	11.15	24.87
	5^th^ Year	14.82**	3.35	<.001	8.24	21.40

* . Significance ≤.05,

** . Significance <.01, SE: Standard Error.

DREEM score analysis according to its domains by medical students of DUHS showed that maximum score was given to 2^nd^ domain of their perception regarding teachers and minimum score was allocated to 5^th^ domain regarding social self-perceptions. Details of all 50 items’ mean scores according to five domains is detailed in Table-IV.

**Table-IV:** Mean scores of 50 items and five domains of Dundee Ready Educational Environment Measure (DREEM) from 1054 medical students of DUHS.

#	DOMAIN ITEMS	Mean (%)	±SD
I.PERCEPTIONS OF LEARNING (Max Score 48)			
1	I am encouraged to participate in class	2.07	1.20
7	The teaching is often stimulating	1.82	1.08
13	The teaching is student centered	1.74	1.24
16	The teaching helps me to develop my competence	1.96	1.21
20	The teaching is well focused	2.00	1.12
22	The teaching is sufficiently concerned to develop my confidence	1.80	1.18
24	The teaching time is put to good use	1.88	1.26
25	The teaching over-emphasizes factual learning	1.85	1.12
38	I am clear about the learning objectives of the program	2.24	1.20
44	The teaching encourages me to be an active learner	1.87	1.23
47	Long-term learning is emphasized over short-term learning	2.08	1.27
48	The teaching is too teacher centered	1.85	1.11
Domain Sub-total		23.16 (48.25%)	14.22
II.PERCEPTIONS OF TEACHERS (Max Score 44)			
2	The teachers are knowledgeable	2.45	1.25
6	The clinicians are patient with patients	2.22	1.04
8	The teachers ridicule the students	2.19	1.15
9	The teachers are authoritarian	1.85	1.06
18	The clinicians have good communications skills with patients	2.35	1.10
19	My social life is good	2.11	1.23
32	The teachers provide constructive criticism here	1.88	1.19
37	The teachers give clear examples	2.06	1.22
39	The teachers get angry in class	2.16	1.21
40	The teachers are well prepared for their classes	2.15	1.26
50	The students irritate the teachers	2.06	1.24
Domain Sub-total		23.47 (53.34%)	12.95
III.ACADEMIC SELF-PERCEPTIONS (Max Score 32)			
5	Learning strategies which worked for me before continuing to work for me now	1.95	1.09
10	I am confident about my passing this year	2.57	1.23
21	I feel I am being well prepared for my profession	1.93	1.19
26	Last year’s work has been a good preparation for this year’s work	2.03	1.08
27	I am able to memorize all I need	1.78	1.21
31	I have learned a lot about empathy in my profession	2.19	1.30
41	My problem-solving skills are being well developed here	1.89	1.18
45	Much of what I have to learn seems relevant to a career	1.82	1.12
Domain Sub-total		16.17 (50.53%)	9.39
IV.PERCEPTIONS OF ATMOSPHERE (Max Score 48)			
11	The atmosphere is relaxed during the clinic teaching	2.10	1.16
12	This college is well timetabled	1.65	1.34
17	Cheating is a problem in this college	2.47	1.35
23	The atmosphere is relaxed during lectures	2.10	1.22
30	There are opportunities for me to develop interpersonal skills	1.91	1.25
33	I feel comfortable in class socially	2.17	1.50
34	The atmosphere is relaxed during seminars/tutorials	2.32	1.27
35	I find the experience disappointing	2.09	1.26
36	I am able to concentrate well	1.89	1.17
42	The enjoyment outweighs the stress of the program	1.71	1.30
43	The atmosphere motivates me as a learner	1.85	1.25
49	I feel able to ask the questions I want	2.13	1.28
Domain Sub-total		24.40 (50.83%)	15.35
V.SOCIAL SELF-PERCEPTIONS (Max Score 28)			
3	There is a good support system for students who get stressed	1.34	1.29
4	I am too tired to enjoy the course	1.62	1.24
14	I am rarely bored on this program	1.65	1.26
15	I have good friends in this college	2.56	1.38
19	My social life is good	2.11	1.23
28	I seldom feel lonely	1.98	1.27
46	My accommodation is pleasant	2.21	1.22
Domain Sub-total		13.47 (48.11%)	8.88

### Students’ perception of learning

Scores of items with <2 points refer to stimulative teaching, student centered teaching, competence & confidence development, good use of time, factual learning atmosphere, active learning & teacher centered teaching environment. This domain was ranked at 4^th^ position with 48.25% in our study.

### Students’ perception of teachers

Items in this domain with score of <2 pertains to authoritarian teachers & constructive criticism. This domain was ranked at 1^st^ position in our study at 53.34%.

### Students’ academic self-perception

Items in this domain with score of <2 pertains to learning strategies, preparedness for profession, memorization, problem-solving & relevance to career. This domain was ranked at 3^rd^ position with a score of 50.53%.

### Students’ perceptions of atmosphere

Items in this domain with scores of <2 pertains to well time-tabled, interpersonal skills, concentration, enjoyment vs stress & motivation. This domain was ranked at 2^nd^ place with a score of 50.83%.

### Students’ social self-perceptions

Items with score of <2 pertains to stress support program, tiredness, boredom & loneliness. This domain was ranked at 5^th^ position with a score of 48.11%.

## DISCUSSION

In our study the mean of total score of DREEM by DMC students was 100.07 and that of DIMC was 100.52, both these values fall into category of ‘many problems’. In our study the highest mean scores for the DREEM inventory were recorded for the first year while minimum scores were given by 4^th^ year students. It has been reported that higher DREEM scores tend to indicate more student-centered curricula, while those offering conventional curricula commonly score less than 120 out of 200.^10^-^12^

The students’ perception can be used to initiate change and improvement in EE of the institute. Evaluation of EE should be part of educational practices by any institute.^13^,^14^ A study showed that when learners feedback was incorporated into curriculum, the learners’ satisfaction level improved significantly.^15^ We need to ensure that the environment is as conducive as possible to learning, thus reducing the risk of academic underachievement. Our results showed that it is essential for faculty members and course managers to make more efforts toward observing principles of instructional designs, to create an appropriate educational environment, and to reduce deficits to provide a better learning environment with more facilities and supportive systems for the students.

In a study from Peshawar, Pakistan comparison were done between pre-clinical and clinical students regarding educational environment and they did not find any difference in opinion.^11^ This scale has been used in many institutions in Pakistan for assessment of student’s opinion.^6^,^15^-^18^ Our results were similar to study conducted in India in which global scores were 101.13 and lowest scores of 89.80 were given by final semester students.^8^ This trend was also observed in our study where highest scores were given by 1^st^ year which gradually decreased to lowest for 4^th^ year, but 5^th^ year students scored slightly better than 4^th^ year students. Same effect was also seen in another study which also showed that the scores given by 1^st^ year were highest and lowest by final year students.^3^,^19^,^20^

### Students’ perception of learning

Low scores of items in this domain refer to stimulative and student-centered teaching. Different approaches could be used leading to decrease in teacher-centered learning methods. Also training teachers on newer teaching and assessment methods could regenerate the interest of students and increase their confidence level. Clinical classes and bedside teaching should be modified to improve the learning experience.^19^

### Students’ perception of teachers

This domain ranked highest in our study showing students’ satisfaction regarding teachers. Items in this domain with score of <2 pertains to authoritarian teachers and provision of constructive criticism. This could be addressed by training teachers to level with students and undertake confidence-building measures.

### Students’ academic self-perception

the low scoring items in this domain were learning strategies currently employed in institute, short group and individual attention could improve the perspective. Explaining concepts in detail will improve memorization and case scenarios will improve problem solving abilities of students.

### Students’ perceptions of atmosphere

students scored low on interpersonal skills; this should be addressed by interpersonal skills workshops made part of curriculum. Students should also be provided with means to reduce stress and stress management and psychiatric help should be available on campus with some motivational lectures.

### Students’ social self-perceptions

This was the most problematic domain and it ranked lowest in our study. Items scoring low in this domain refers to stress support, tiredness, boredom and loneliness. These are all part of stress management and factors inducing depression in students. Steps to improve on these items are important not only to reduce stress in students but also to decrease incidence of depression and associated problems in students. Entertainment for students could be provided by indoor games and media streaming in common rooms.

In many studies that used DREEM for EE, it was found that Q3 (There is a good support system for students who get stressed) was found to be most problematic. This was followed by Q27 (I am able to memorize all I need), Q4 (I am too tired to enjoy the course), Q9 (Teachers are authoritarian), Q25 (The teaching overemphasizes factual learning), Q42 (The enjoyment outweighs the stress of the course), and Q48 (The teaching is too teacher oriented).^21^ The items of concern in our study were 3, 4, 5,7, 9, 12, 13, 14, 16, 21, 22, 24, 25, 27, 28, 30, 32, 36, 41, 42, 43, 44, 45 & 48. Out of these Q3 (There is a good support system for students who get stressed), Q9 (The teachers are authoritarian), Q12 (This college is well timetabled) need urgent redressal by institute administration. In comparison with some other institutes where Q17 (Cheating is a problem in the school) and Q39 (The teachers get angry in the class) were not identified as issues in our study and DUHS is managing these items amenably.

Five items that got highest scores in our study in descending order are Q10 (I am confident about my passing this year; 2.57 ±1.23), Q15 (I have good friends in this college; 2.56 ±1.38), 17 (Cheating is a problem in this college; 2.47 ±1.35), Q2 (The teachers are knowledgeable; 2.45 ±1.25) & Q18 (The clinicians have good communications skills with patients; 2.35 ±1.10). Students’ self-opinion regarding their passing is a good omen more over students also highly ranked teachers’ knowledge and clinicians’ good communications skills with patients. Also, in students’ opinion cheating is not a problem in DUHS.

Five items that got lowest scores in ascending order were Q3 (There is a good support system for students who get stressed; 1.34 ±1.29), Q4 (I am too tired to enjoy the course; 1.62 ±1.24), Q12 (This college is well timetabled; 1.65 ±1.34), Q14 (I am rarely bored on this program; 1.65 ±1.26) & Q42 (The enjoyment outweighs the stress of the program; 1.71 ±1.30).

University management needs to address the low scoring domains for better student experience in university that will also increase their interest in education.

### Limitations

It was conducted in only one university.

## CONCLUSION

Students perceived EE in DUHS as “many problems”. Some negative aspects were also revealed, e.g., lack of support for students who are stressed, more tried to enjoy course, poor timetable and getting bored. These facts need looking into for betterment of EE. A change in attitudes and approach by teachers and administration will mitigate the deficiencies pointed out by students and will help to provide better EE for students thus helping them to become very competent professionals.

### Author`s Contribution:

**BFZ:** Conceived the study idea & did final approval of manuscript.

**MAS:** Prepared the manuscript.

**FFZ:** Did data collection, edited/corrected manuscript & responsible to integrity of data.

**TZ:** Did data collection, edited/corrected manuscript.

**FSA:** Did statistical analysis.

**ZN:** Did data collection, edited/corrected manuscript & responsible to integrity of data.

## References

[1] Thomas PA, Kern DE, Hughes MT, Chen BY (2016). Curriculum Development for Medical Education:A Six-Step Approach. JHU Press.

[2] Jawaid M, Ashraf J (2012). Initial Experience of Elearning Research Module in Undergraduate Medical Curriculum of Dow University of Health Sciences:Development and Students Perceptions. Pak J Med Sci.

[3] Ranade AR, Jadhav AJ, Wagh SY (2023). Comparative Analysis of Education Environment Perception of 1 St and 2^nd^ Year MBBS Students of Medical College Using Dundee Ready Education Environment Measure. Asian J Med Sci.

[4] Seco-Calvo J, Lantaron E, Martinez J, Escobar G, Esteve E, Franco-Sierra M (2023). Spanish Version of the Dundee Ready Education Environment Measure (Dreem) Applied to Undergraduate Physical Therapy Students in Spain Using Google Form. Med Teach.

[5] Dow University of Health Sciences:Integrated Modular Curriculum [PDF] (2016). Karachi:DUHS.

[6] Jawaid M, Raheel S, Ahmed F, Aijaz H (2013). Students'Perception of Educational Environment at Public Sector Medical University of Pakistan. J Res Med Sci.

[7] Kavukcu E, Burgazli KM, Akdeniz M, Bilgili P, Oner M, Koparan S, Yorumez A (2012). Family Medicine and Sports Medicine Students'Perceptions of Their Educational Environment at a Primary Health Care Center in Germany:Using the Dreem Questionnaire. Postgrad Med.

[8] Kohli V, Dhaliwal U (2013). Medical Students'Perception of the Educational Environment in a Medical College in India:A Cross-Sectional Study Using the Dundee Ready Education Environment Questionnaire. J Educ Eval Health Prof.

[9] Shrestha B, Dunn L (2020). The Declaration of Helsinki on Medical Research Involving Human Subjects:A Review of Seventh Revision. J Nepal Health Res Counc.

[10] Latif M, Wajid G (2018). Reforming Medical Education in Pakistan through Strengthening Departments of Medical Education. Pak J Med Sci.

[11] Raza A, Khaliq T (2022). Students Dreem from Pre-Clinical to Clinical Years-a Cross Sectional Analysis from Rehman Medical College, Peshawar. J Ayub Med Coll Abbottabad.

[12] Al-Ahmari MM, Al Moaleem MM, Khudhayr RA, Sulaily AA, Alhazmi BAM, AlAlili MIS (2022). A Systematic Review of Publications Using the Dundee Ready Education Environment Measure (Dreem) to Monitor Education in Medical Colleges in Saudi Arabia. Med Sci Monit.

[13] Kasemy ZA, Kabbash I, Desouky D, El-Raouf SA, Aloshari S, El Sheikh G (2022). Perception of Educational Environment with an Assessment of Motivational Learning Strategies and Emotional Intelligence as Factors Affecting Medical Students'Academic Achievement. J Educ Health Promot.

[14] Essa M (2023). Learning Environment in Faculty of Medicine at Jazan University;a Student Perspective. Hail J Health Sci.

[15] Ahmed SD, Mubeen SM (2013). Exploring Teaching Style in an Undergraduate Medical College Following Traditional Curriculum in Pakistan. J Pak Med Assoc.

[16] Rehman R, Ghias K, Fatima SS, Hussain M, Alam F (2017). Dream of a Conducive Learning Environment:One Dreem for All Medical Students!. J Pak Med Assoc.

[17] Mushtaq R, Ansar A, Bibi A, Ramzan M, Munir A, Zaheer A (2017). Quality of Educational Environment at Wah Medical College:Assessment by Using Dundee Ready Educational Environment Measure. J Ayub Med Coll Abbottabad.

[18] Rathore FA, Waqas A, Zia AM (2016). A Thirteen Year Audit of Manuscripts Related to Medical Education Published in Leading Medical Journals of Pakistan. J Pak Med Assoc.

[19] Chandran CR, Ranjan R (2015). Students'Perceptions of Educational Climate in a New Dental College Using the Dreem Tool. Adv Med Educ Pract.

[20] John J, Thomas S, Michael ME, Thomas NL, Paul P (2023). Perception of Nursing Students Towards Their Academic and Clinical Learning Environment. Res J Sci Technol.

[21] Foster Page LA, Kang M, Anderson V, Thomson WM (2012). Appraisal of the Dundee Ready Educational Environment Measure in the New Zealand Dental Educational Environment. Eur J Dent Educ.

